# Research to improve differentiated HIV service delivery interventions: Learning to learn as we do

**DOI:** 10.1371/journal.pmed.1002809

**Published:** 2019-05-21

**Authors:** Elvin H. Geng, Charles B. Holmes

**Affiliations:** 1 Department of Medicine, University of California San Francisco, San Francisco, California, United States of America; 2 Center for Global Health and Quality, Georgetown University Department of Medicine, Washington, DC, United States of America; 3 Johns Hopkins School of Medicine, Baltimore, Maryland, United States of America

## Abstract

In a Perspective, Elvin Geng and Charles Holmes discuss research on differentiated service delivery in HIV care.

Many low- and middle-income countries with high HIV prevalence are working to modernize their approaches to HIV treatment service delivery. Under the umbrella term of “differentiated service delivery” (DSD), health systems are evolving away from traditional models of care, in which encounters were both frequent and exclusively at the health facility, to models that vary the location (e.g., to venues in the community), reduce the frequency (e.g., quarterly, biannually), and expand the roles of pharmacists, nurses, lay healthcare workers, and patients themselves (e.g., for peer drug distribution) in service delivery [1]. Diverse practices from increasing antiretroviral medications dispensed at each visit (and thereby reducing visit frequency), to community groups where one member picks up medications for all, and many others are considered DSD models. DSD models, however, are unified by reducing unnecessary barriers to seeking care for individual patients as well as relieving frail health systems of clinically unnecessary patient encounters—which, in turn, enables reallocation of resources to reach even more patients.

The role of research in the global scale-up of DSD models is a topic of sometimes heated discussion. Although some suggest that rigorous research is required before fully investing in scale-up of DSD, that view imposes, in our view, an unreasonable standard for innovation. After all, the status quo treatment models with highly standardized encounter intervals, irrespective of an individual’s clinical condition and treatment history, emerged when antiretroviral therapy (ART) was restricted and many patients had low CD4 levels and relatively advanced immunosuppression. Today, because of treatment for all and a growing time on treatment, most patients are healthy, and practices targeting the sickest individuals represent an unnecessary burden for the typical patient. On the other hand, adoption of strategies without accompanying research fails to recognize the contributions that rigorous causal knowledge can play. Different models make different demands on the health system and also reduce barriers to care through different mechanisms (i.e., simply dispensing more medications at each visit addresses opportunity costs but does not create the social support that is hypothesized to play a role in models with patient groups)—which implies that their roles may differ across contexts. Understanding the comparative implementability and effectiveness (especially in combination) of DSD models through research will inform strategies for continued scalability and anticipate sustainability. Investing in implementation-based evidence during scale-up of DSD will strengthen progress toward evidence-based implementation.

In this issue of *PLOS Medicine*, Colleen Hanrahan and colleagues present research on DSD models at a moment when many countries in Africa are already implementing and expanding them. At a single primary care clinic setting in northern Johannesburg, South Africa, they enrolled adults on ART and with undetectable HIV plasma RNA levels [2] and randomized participants to either a community- or facility-based adherence group—groups of 25–30 persons who met at a designated time every other month to pick up medications and provide an opportunity for social support. Those who missed a club visit without ART pickup within five days, had two consecutive late ART pickups, developed a comorbidity requiring closer monitoring, or had viral rebound were referred back to usual care. Of 775 patients randomized (65% female, median CD4 count of 506/μl), at 24 months, 57% in facility-based clubs remained in the clubs, while only 48% in the community-based clubs were retained in the club model, and overall retention (which included retention in any care) was 93% and 88%, respectively. Both differences were statistically significant. HIV plasma RNA suppression rates were not reported. In short, patients fared slightly better in clinic-based clubs than community-based clubs, but persistence in either club model was poor, and retention after return to usual care was high.

When viewed in the context of the evolution of DSD models, these main findings sound an important and credible dissenting note to a budding conviction that treatment in the community is better than in the facility. Community-based treatment was considered early in the global treatment response, but enthusiasm for this approach grew in 2011 when a study from Mozambique found that patients who joined self-forming groups of six (i.e., “community adherence groups”), who rotated visits to clinics and collected drugs for the other group members, achieved extraordinary levels of retention (98% at a time when other studies were reporting around 60% retention) [3]. These data, in a setting of overcrowded facilities, overwhelmed providers, and extended waiting times [4], led some to believe that community-based models in general represented a better strategy. Hanrahan’s randomized design protects their findings against the principal bias incurred by previous observational studies—specifically, the selection of patients most likely to succeed into specific models of care—and therefore suggests that facility-based models are not necessarily always inferior to, and indeed in some cases may be better than, services delivered in the community.

In addition to the main findings, Hanrahan's study offers other useful insights for policy makers and planners who have committed to use of DSD models but must now decide which models to prioritize. Specifically, while Hanrahan’s study found small differences in effectiveness (as measured by retention) between community-based and facility-based clubs, retention after return to usual care appeared to be better than in either club model (even though this was not the randomized comparison). The relatively low sustained retention in club-based care here complements recent data from Malawi, in which patient group models (i.e., community adherence groups) experienced relatively low enrollment [5]. While more research to understand patient concerns about club-based treatment could be revealing [6], one conclusion could be that where the supply chain can support it, simply giving 3 or 6 months of medications at each visit can be as or more effective compared to club-based models in alleviating the burden associated with seeking care while demanding less supervision, coordination, and organizational change to monitor or tend to groups.

Despite its contributions, Hanrahan’s study also illustrates common perils in current conduct of implementation science. First, Hanrahan’s study is implementation science regardless of whether the authors claim that category—adherence clubs (an implementation strategy) seek to enhance the sustained use of ART (an evidence-based intervention)—and is therefore destined to be appraised through this lens. First, by randomizing individuals to two models, the results are not directly interpretable in a plausible future reality in which different models are offered and patients simply select the model they prefer. Implementation studies must double down on choice when choice is likely to be a feature in target routine care settings. It could be argued that in some situations patient preference is the sole issue at hand, in which case a choice experiment would have saved much expense and effort. Second, understanding the mechanism is as important as understanding effects because effects (in implementation science) are often contextual, and mechanisms inform generalizability or external validity. Why did the patients stop attending club visits? What was the median visit schedule after return to clinic in standard of care? What was the role of stigma? The answers to these questions are needed to understand why the membership in the clubs fell precipitously and why membership fell more than in standard care. Third, the inflexibility of the study intervention (e.g., the strict rules guiding dismissal from group care) weakens the findings. Inflexible interventions do not automatically confer rigor, and if excessive rigidity is not possible at scale, neither should it be imposed in a pragmatic research study. The authors argue in their discussion that retention in the clubs was not high enough to justify the resources used to run the clubs. An alternative interpretation could be that clubs, whether community or facility, may have been an optimal and patient-centered choice for the roughly half of individuals who wanted them and therefore stayed in them—resource, program, and policy questions worth pursuing further.

Amara’s “First Law of Technology” states that we tend to overestimate the effect of a technology (or innovation) in the short run and underestimate the effect in the long run. DSD models are no panacea for complex issues in global health systems but do represent a strategic pillar of the HIV response likely to grow in impact over time. Hanrahan’s study reminds us not only of the potential contributions that research can make but also of the need for the right research at the right time in the right context. Rigorous and relevant implementation research can accelerate the transition in Amara’s Law from short-term hype to realization of our long-term collective objectives to end human suffering from the HIV pandemic and beyond.
